# The impact of thermal pre-conditioning on cutaneous vasomotor and shivering thresholds

**DOI:** 10.1186/2046-7648-4-S1-A117

**Published:** 2015-09-14

**Authors:** Joanne N Caldwell, Åsa Nykvist, Nicholas Powers, Sean R Notley, Daniel S Lee, Gregory E Peoples, Nigel AS Taylor

**Affiliations:** 1 Centre for Human and Applied Physiology, School of Medicine, University of Wollongong, Wollongong, Australia

## Introduction

The mean body temperature of resting, normothermic humans falls within the zone that separates the temperature thresholds for shivering and sweating; the vasomotor zone. Whilst these thresholds are often defined by their corresponding deep-body or mean body temperatures, it is well known that these are not set temperatures or points. Nevertheless, our knowledge concerning the factors that determine or modify these thresholds is imprecise. Therefore, the aim of this experiment was to investigate the effects of a deliberate modification of the pre-exposure mean body temperature on the subsequent vasomotor and shivering threshold temperatures. Mean body temperature was first displaced upwards, then slowly driven in the opposite direction, permitting the separate determination of these thermoeffector thresholds.

## Methods

Eight males participated in two trials, each consisted of a pre-experimental, whole-body water immersion followed by passive cooling (experimental phase). Trials differed in the pre-experimental water temperature (45 min), with subjects either maintained in a normothermic (control: 34 °C) or a heated state (39 °C). Following pre-treatment and thermal clamping, subjects were passively cooled (water-perfusion garment) until 10 min beyond the onset of overt shivering. Deep-body and skin temperatures were recorded continuously, with skin blood flows (finger, forearm, calf; venous-occlusion plethysmography) and shivering (whole-body oxygen consumption) recorded during the experimental phase.

## Results

Prior to cooling, mean body temperatures averaged 35.12 °C (±0.2; control) and 36.74 °C (±0.3; *P *< 0.05). The rate of passive cooling (mean body temperature) was not different between trials: 2.16°C.h^-1 ^(±0.31; control) and 2.15°C.h^-1 ^(±0.21; *P *> 0.05). In the control trial, each vasoconstrictor threshold occurred at a significantly higher mean body temperature than shivering. This was also evident following pre-heating, but now with each threshold being significantly elevated relative to the control trial. Furthermore, the shivering threshold displacement and the change in the pre-exposure mean body temperature were of an equivalent magnitude (*P *> 0.05).

## Discussion

Given the level of experimental control, these threshold changes cannot be associated with between-trial differences in cooling rate. Instead, these observations are consistent with the possibility that thermoeffector thresholds may be as dependent upon the change in mean body temperature as they are upon its absolute value. Indeed, in a previous communication to this society [1], it was demonstrated that the sudomotor threshold was similarly linked with the size of the change in mean body temperature induced prior to commencing the experiment.
